# Supplementary material for: Selective Anticancer and Antimicrobial Metallodrugs Based on Gold(III) Dithiocarbamate Complexes
Source: Biomedicines. 2021 Nov 26;9(12):1775. doi: 10.3390/biomedicines9121775 (PMC8698672; doi:10.3390/biomedicines9121775)
Supplement: Supplementary file 1 [file biomedicines-09-01775-s001.zip › biomedicines-1443493-supplementary.pdf]

# Supporting Information

**for**

# Selective anticancer and antimicrobial metallodrugs based on gold(III) dithiocarbamate complexes

**Elisa Abás [1,2*], Diego Aguirre-Ramírez [2], Mariano Laguna [1] and Laura Grasa [2,3,4*]**

1 Departamento de Química Inorgánica
Instituto de Síntesis Química y Catálisis Homogénea, Universidad de Zaragoza-CSIC.
Plaza S. Francisco s/n, 50009, Zaragoza, Spain
E-mail: 113elisa@gmail.com
2 Departamento de Farmacología, Fisiología y Medicina Legal y Forense, Facultad de Veterinaria.
Universidad de Zaragoza,
Miguel Servet, 177, 50013, Zaragoza, Spain
E-mail: lgralo@unizar.es
3 Instituto de Investigación Sanitaria de Aragón (IIS Aragón)
4 Instituto Agroalimentario de Aragón -IA2- (Universidad de Zaragoza-CITA),

* Correspondence: lgralo@unizar.es, 113elisa@gmail.com

**Table of Contents**

**Figure S1.** $^{1}$H-NMR spectra of **C1**-**6** complexes, and APT spectra for **C3**, **C5**-**C6.**

**Figure S2.** Comparison of the theoretical and experimental MS spectra of complexes **C2**, **C3** and **C6** (from left to right). The software used to predict the theoretical isotopic distribution of the complexes was Bruker Compass Data Analysis 4.0.

**Figure S3.** UV-Vis spectra of **C1**-**6** complexes recorded at different times for 24 h in PBS at 37ºC.

**Figure S4.** UV-Vis spectra of **C1**-**6** complexes recorded at different times for 24 h in PBS at 37ºC in presence of GSH (0-50 µM).

**Figure S5.** UV-Vis spectra of **C1**-**6** complexes recorded at different times for 24 h in PBS at 37ºC in presence of AsA (0-20 µM).

**Figure S6.** Fluorescence emission spectra of BSA at 298 K in the presence of increasing amounts of the gold(III) complexes.

**Figure S7.** Cell viability of tumor Caco-2 cells (5 days after seeding) treated with **C1**-**6** gold(III) complexes for 72h. All the results are expressed as mean ± SEM (n ≥ 12 experiments). *P<0.05; **P<0.01; ***P<0.001 vs. control.

**Figure S8.** Cell viability of normal Caco-2 cells (15 days after seeding) treated with **C1**-**6** gold(III) complexes for 72h. All the results are expressed as mean ± SEM (n ≥ 12 experiments). *P<0.05; **P<0.01; ***P<0.001 vs. control.

**Figure S9.** Fluorescence histograms of the distribution of cell populations in different stages: necrosis (B+-), living cells (B--), late apoptosis (B++) and early apoptosis (B+-), in control (DMSO) and gold(III) complexes (20 µM, 24 h) treated Caco-2/TC7 cells.

**Figure S10.** Fluorescence histograms obtained by flow cytometry of the cell populations in different phases of the cell cycle after 24 h of incubation of the cells with DMSO (control) and gold(III) complexes **1** and **5**−**6** (20 µM).

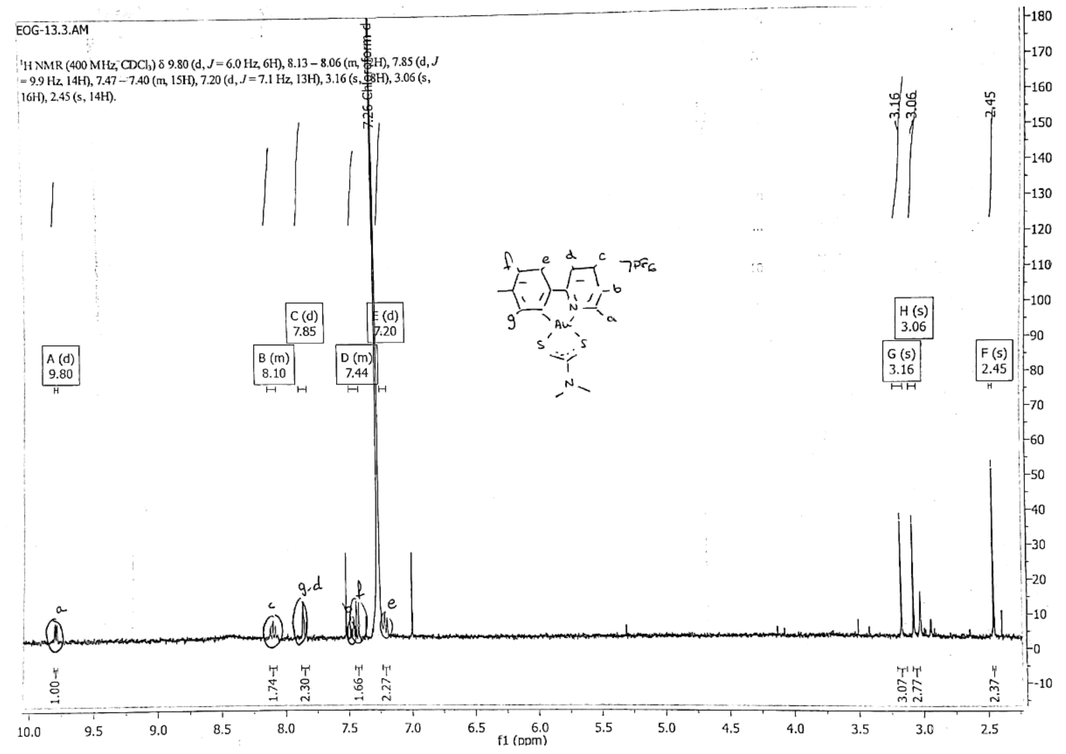

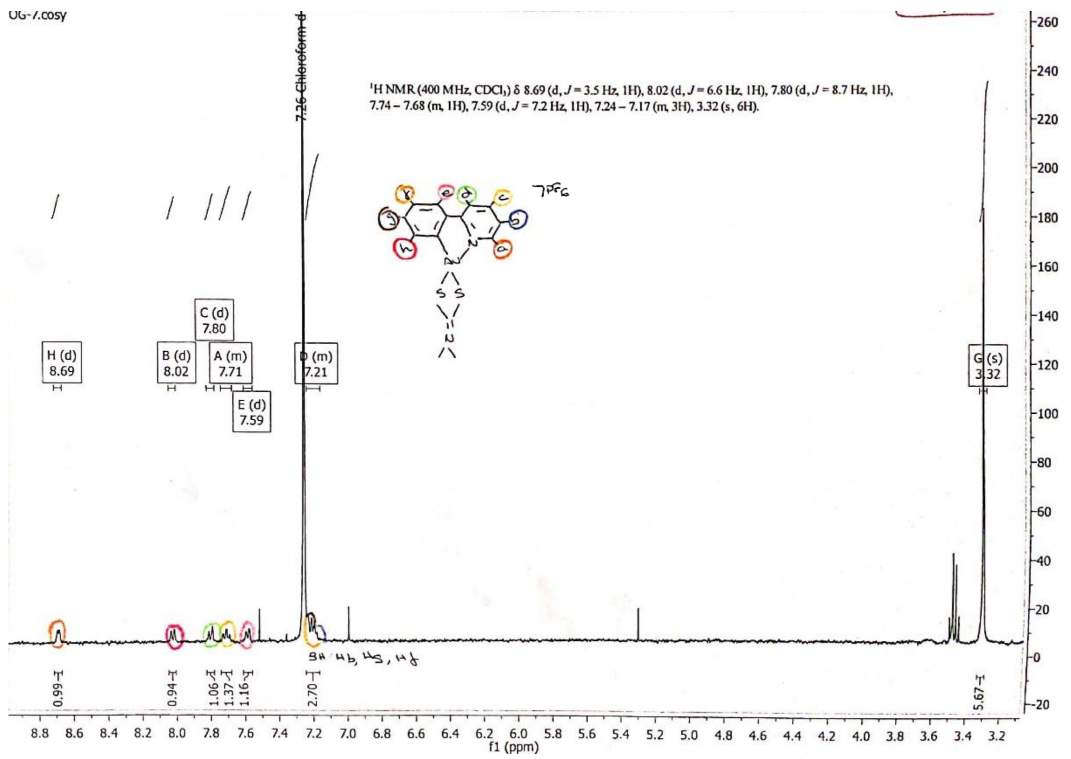

**Figure S1a.** $^{1}$H-NMR spectra of **C1** (above) and **C2** (bottom) complexes in chloroform-d.

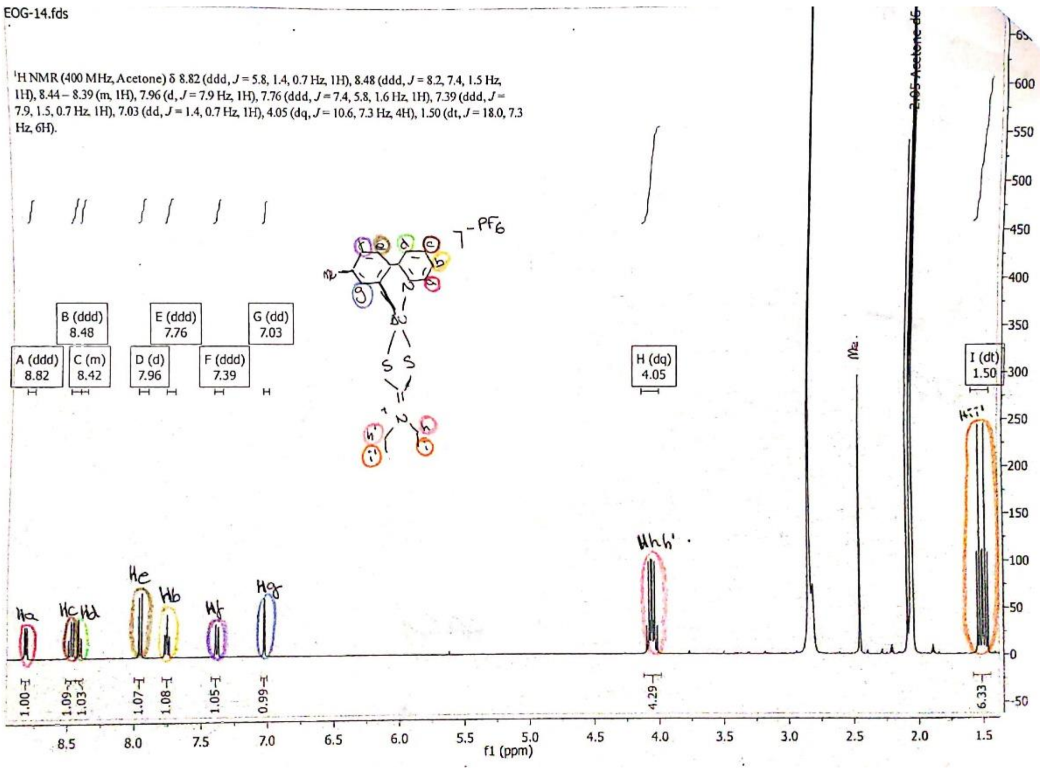


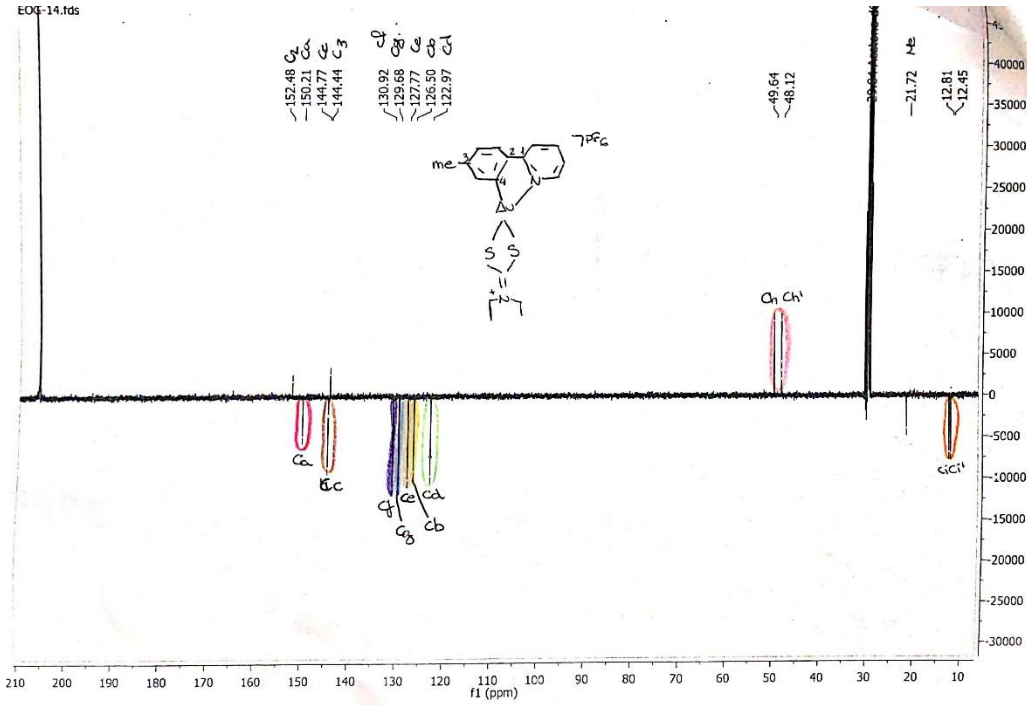


**Figure S1b.** 1H-NMR and APT-NMR spectra of **C3** in acetone-$d_6$.

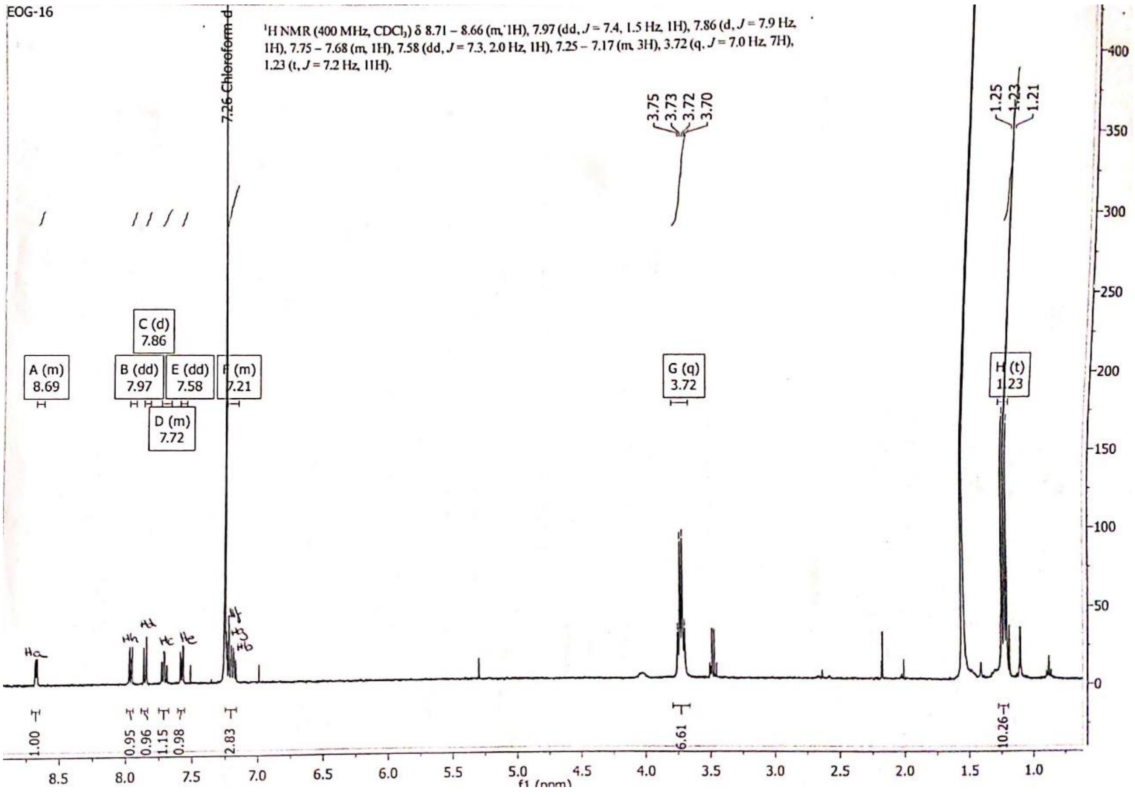


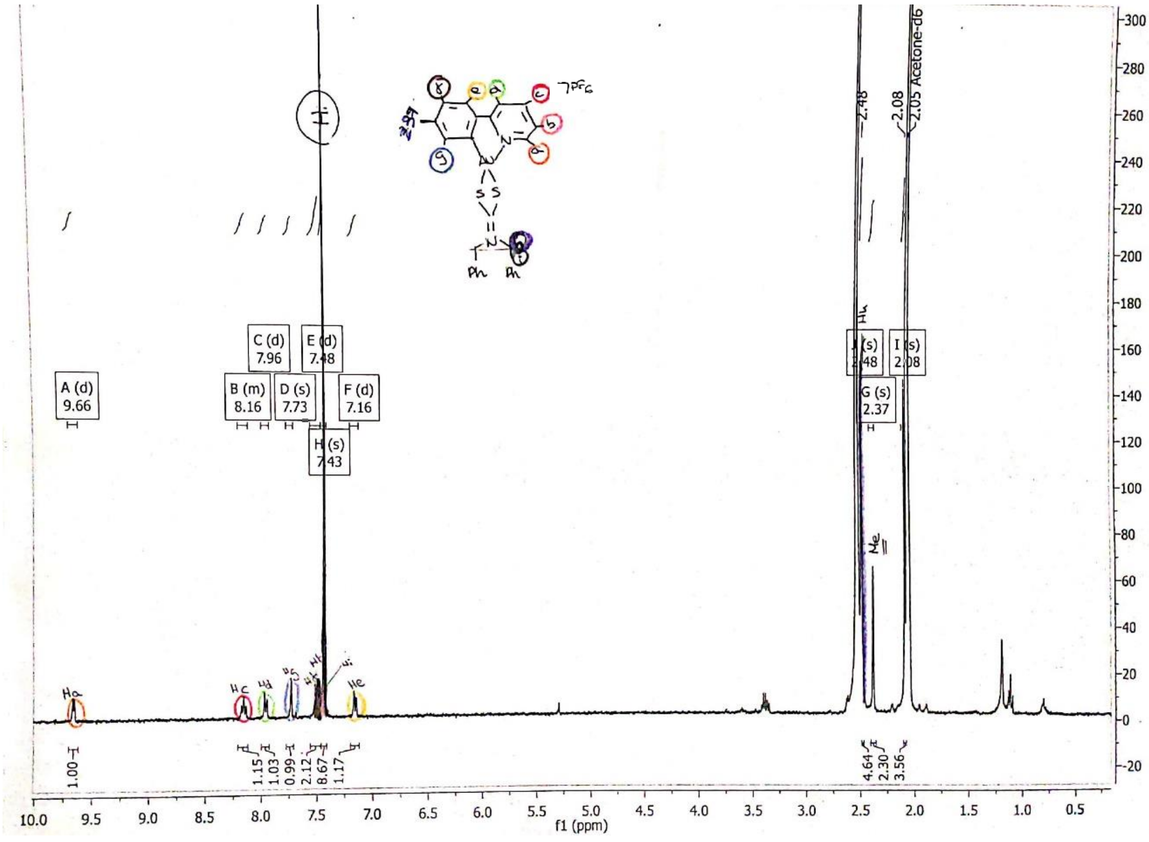


**Figure S1c.** 1H-NMR spectra of **C3** in chloroform-d (above), and **C4** in acetone-$d_6$ (bottom) complexes.

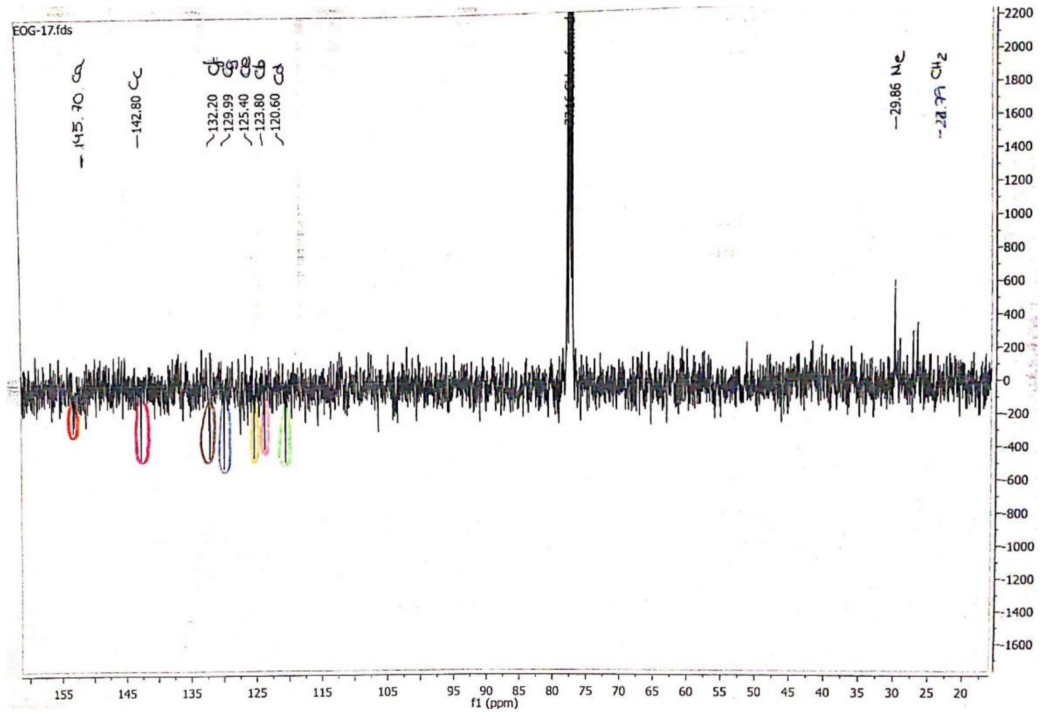

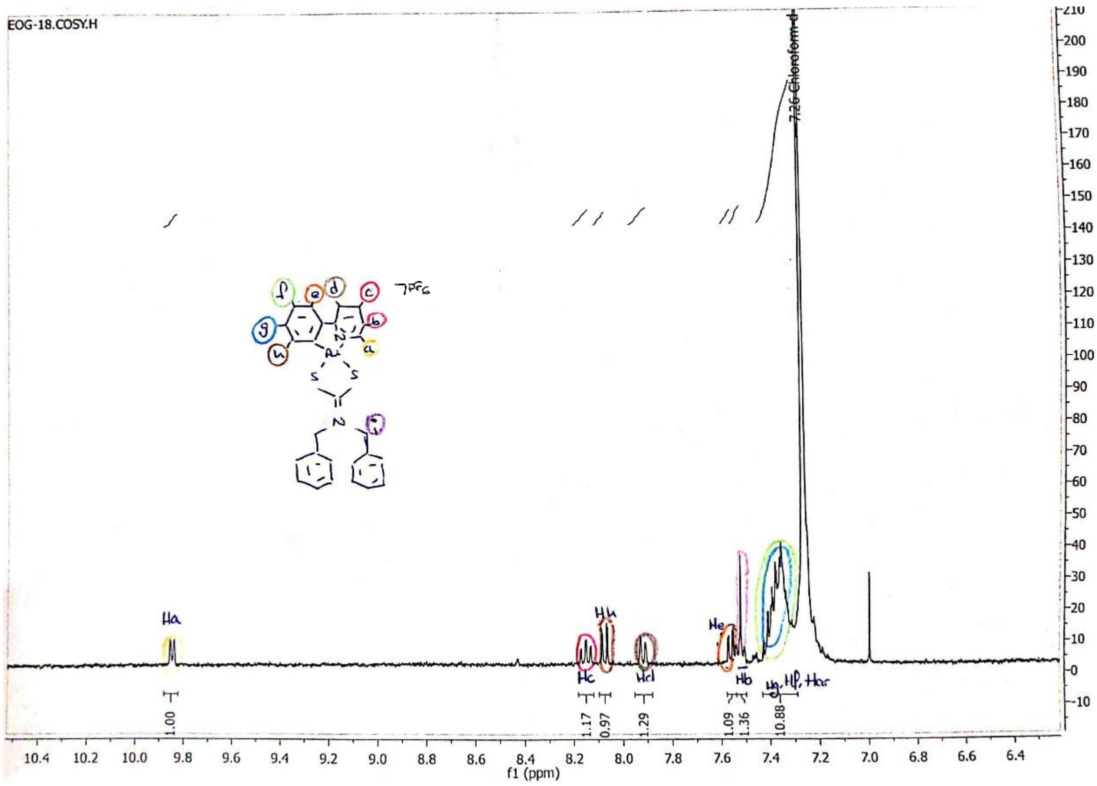

**Figure S1d.** APT-NMR spectrum of **C4** in acetone-$d_6$ (above) and 1H-NMR spectrum of **C3** in chloroform-d (bottom).

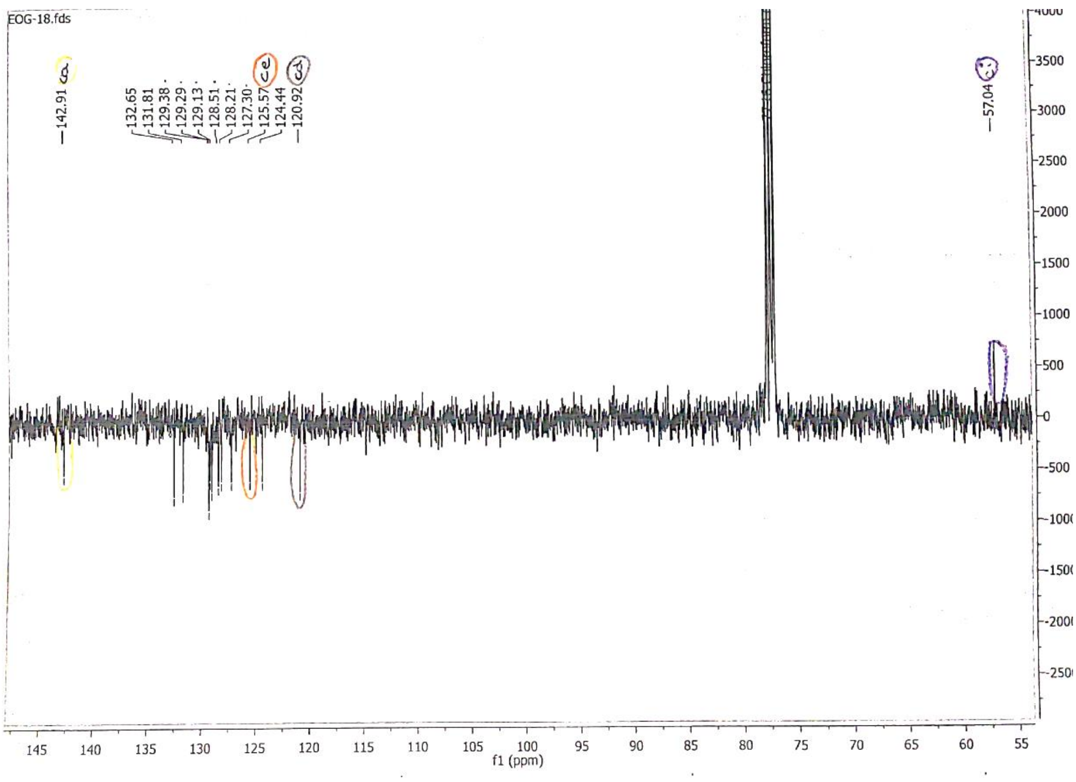


**Figure S1e.** APT-NMR spectrum of **C3** in chloroform-d.

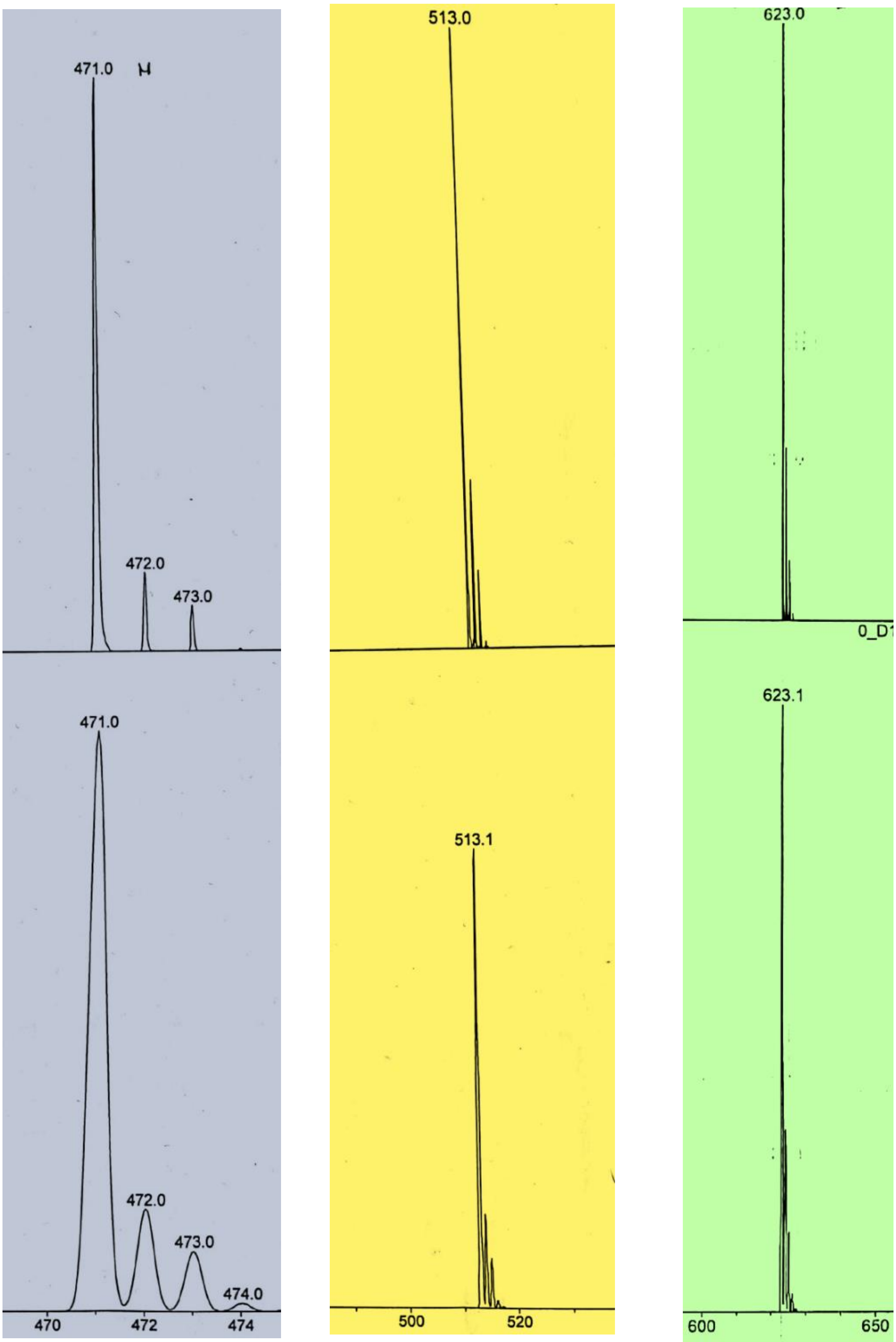


**Figure S2.** Comparison of the theoretical and experimental MS spectra of complexes **C2**, **C3** and **C6** (from left to right).

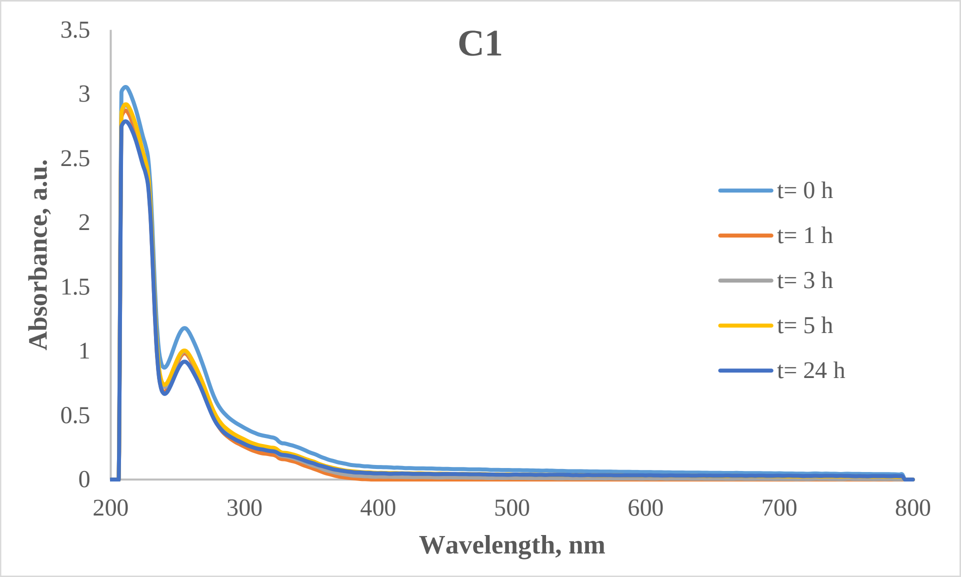
C1
Absorbance, a.u.
3.5
3
2.5
2
1.5
1
0.5
0
200
300
400
500
600
700
800
Wavelength, nm
t= 0 h
t= 1 h
t= 3 h
t= 5 h
t= 24 h

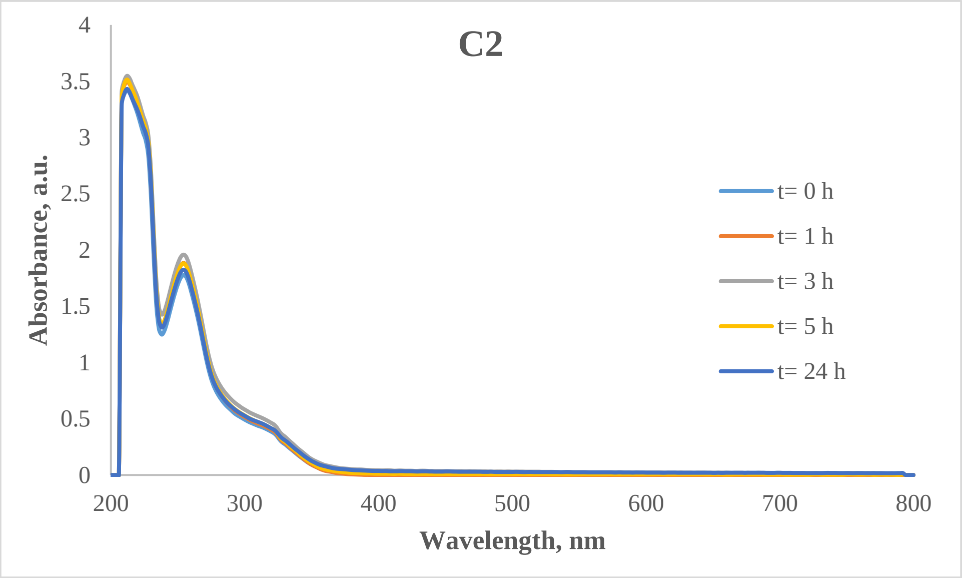
C2
Absorbance, a.u.
4
3.5
3
2.5
2
1.5
1
0.5
0
200
300
400
500
600
700
800
Wavelength, nm
t= 0 h
t= 1 h
t= 3 h
t= 5 h
t= 24 h

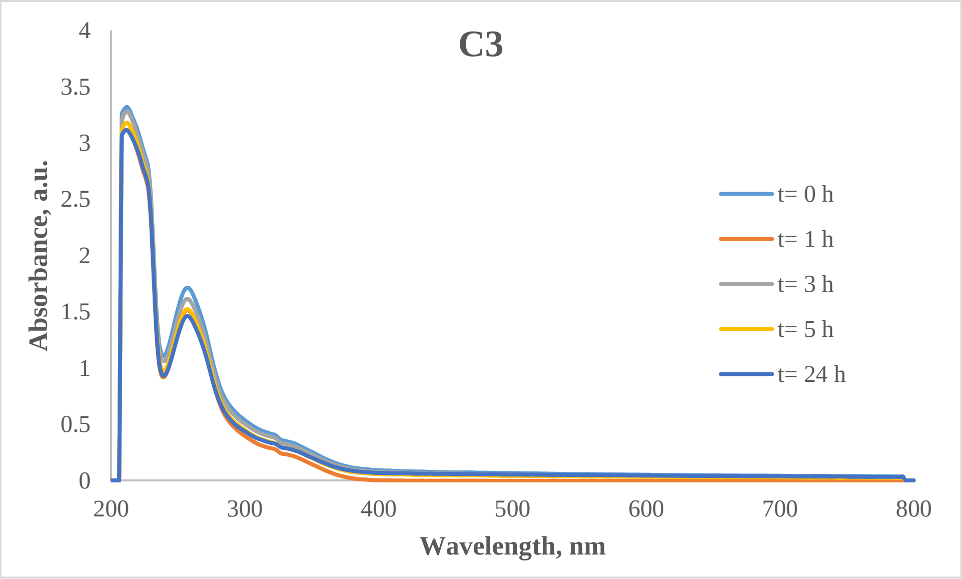
C3
Absorbance, a.u.
4
3.5
3
2.5
2
1.5
1
0.5
0
200
300
400
500
600
700
800
Wavelength, nm
t= 0 h
t= 1 h
t= 3 h
t= 5 h
t= 24 h

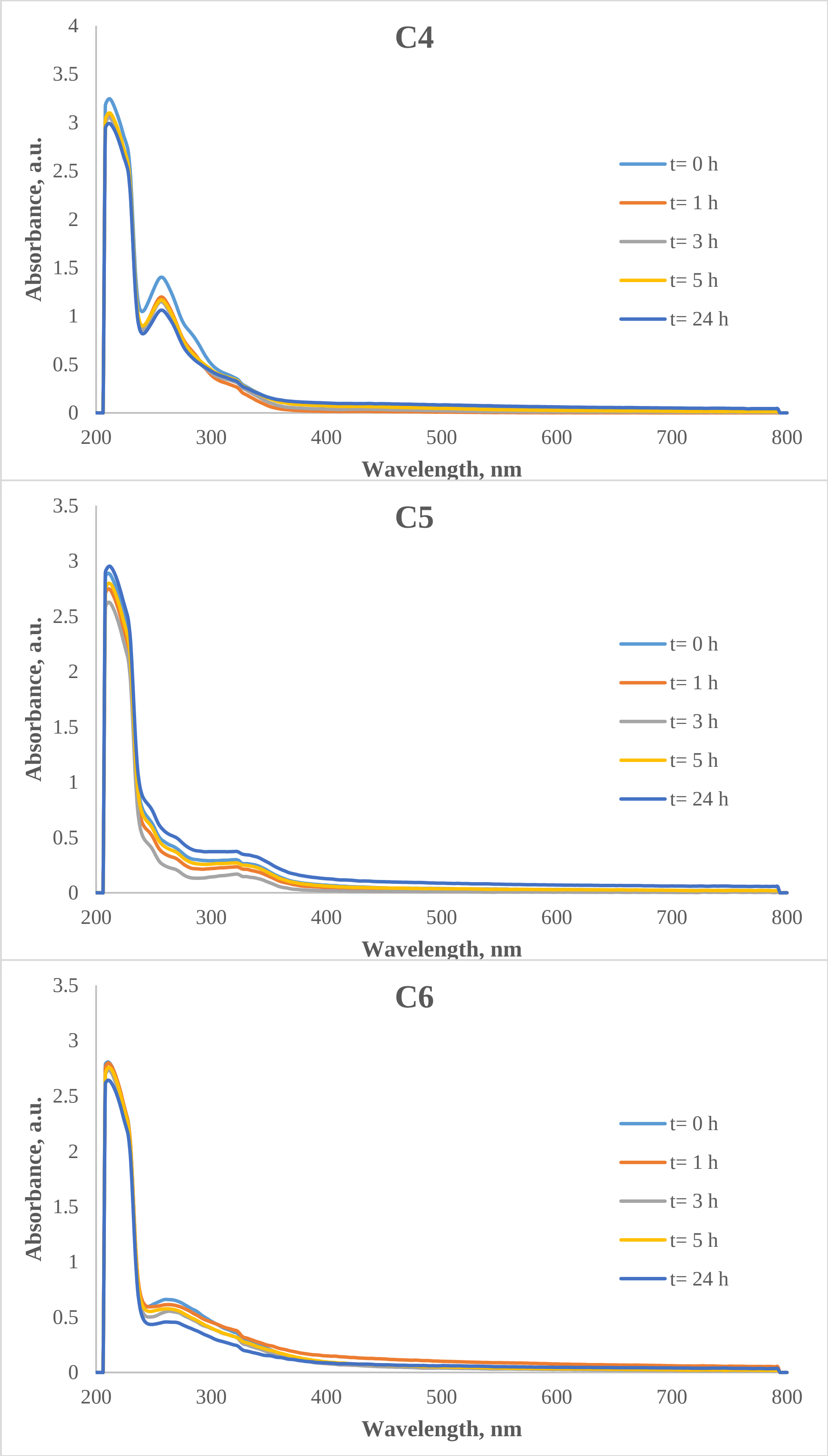


**Figure S3.** UV-Vis spectra of **C1**-**6** complexes recorded at different times for 24 h in PBS at 37ºC.

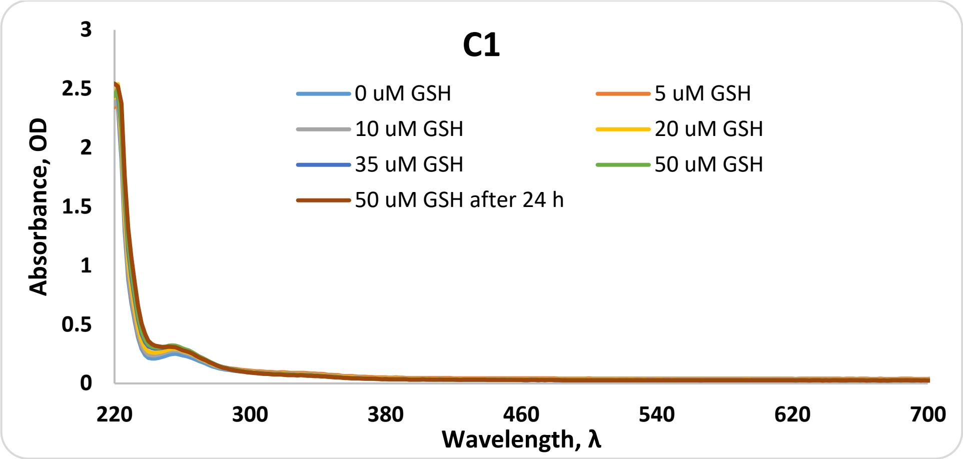
C1
0 uM GSH
5 uM GSH
10 uM GSH
20 uM GSH
35 uM GSH
50 uM GSH
50 uM GSH after 24 h
Absorbance, OD
0
0.5
1
1.5
2
2.5
3
220
300
380
460
540
620
700
Wavelength, λ

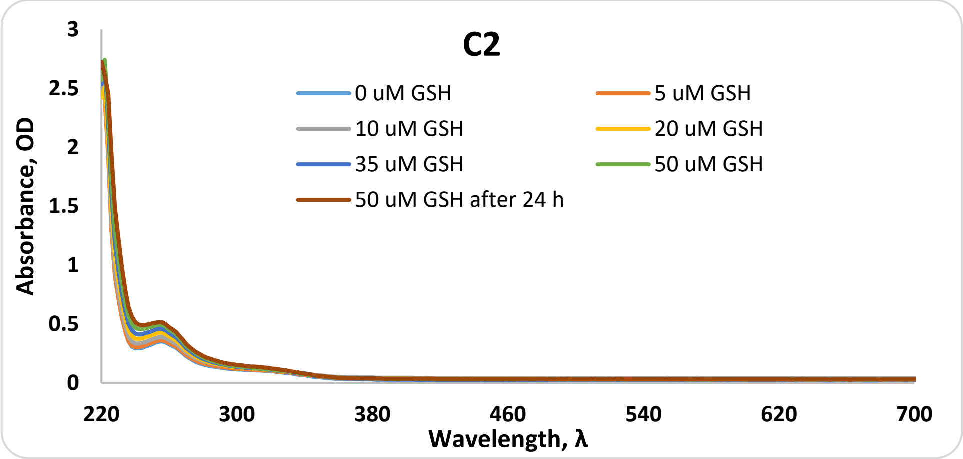
C2
0 uM GSH
5 uM GSH
10 uM GSH
20 uM GSH
35 uM GSH
50 uM GSH
50 uM GSH after 24 h
Absorbance, OD
0
0.5
1
1.5
2
2.5
3
220
300
380
460
540
620
700
Wavelength, λ

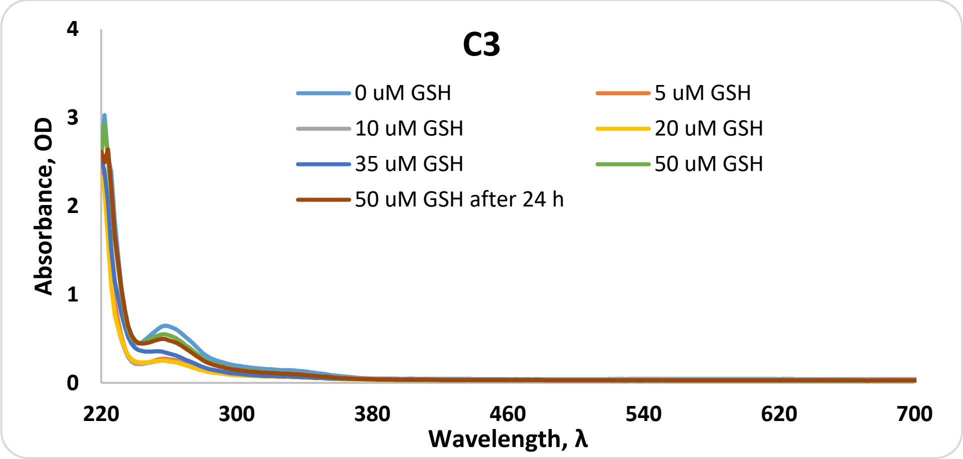
C3
0 uM GSH
5 uM GSH
10 uM GSH
20 uM GSH
35 uM GSH
50 uM GSH
50 uM GSH after 24 h
Absorbance, OD
0
1
2
3
4
220
300
380
460
540
620
700
Wavelength, λ

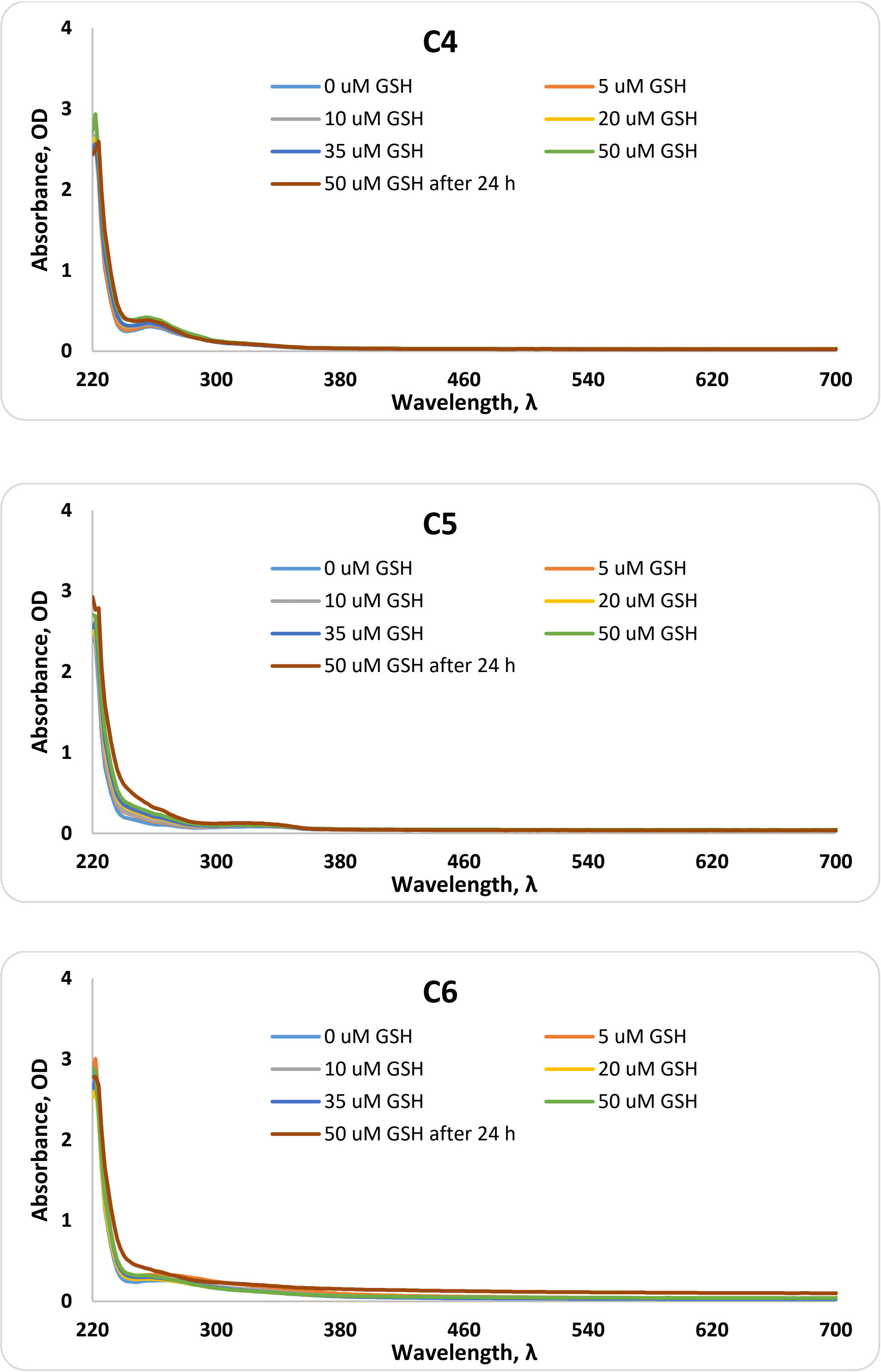


**Figure S4.** UV-Vis spectra of **C1-6** complexes recorded at different times for 24 h in PBS at 37ºC in presence of GSH (0-50 µM).

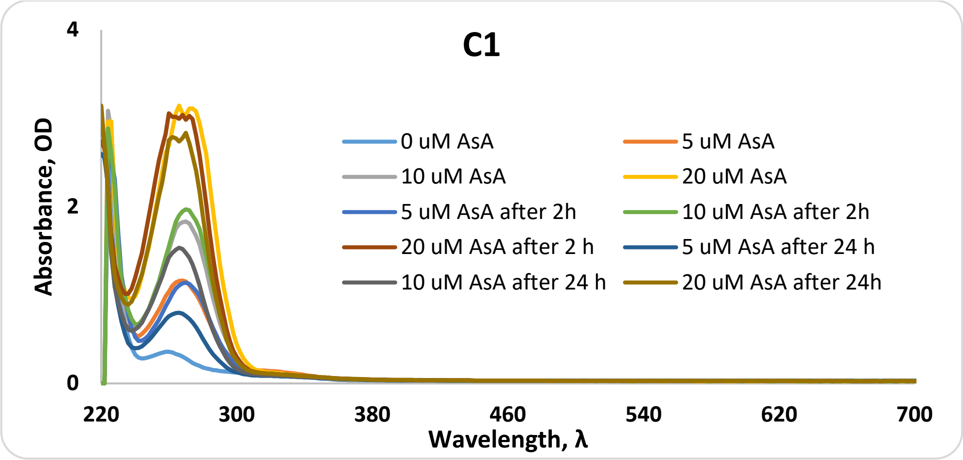
C1
Absorbance, OD
Wavelength, λ
0 uM AsA
5 uM AsA
10 uM AsA
20 uM AsA
5 uM AsA after 2h
10 uM AsA after 2h
20 uM AsA after 2 h
5 uM AsA after 24 h
10 uM AsA after 24 h
20 uM AsA after 24h

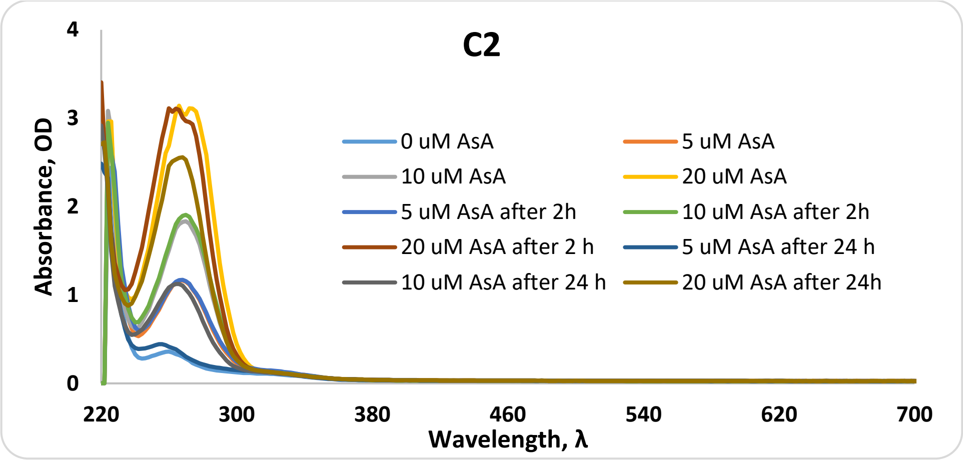
C2
Absorbance, OD
Wavelength, λ
0 uM AsA
5 uM AsA
10 uM AsA
20 uM AsA
5 uM AsA after 2h
10 uM AsA after 2h
20 uM AsA after 2 h
5 uM AsA after 24 h
10 uM AsA after 24 h
20 uM AsA after 24h

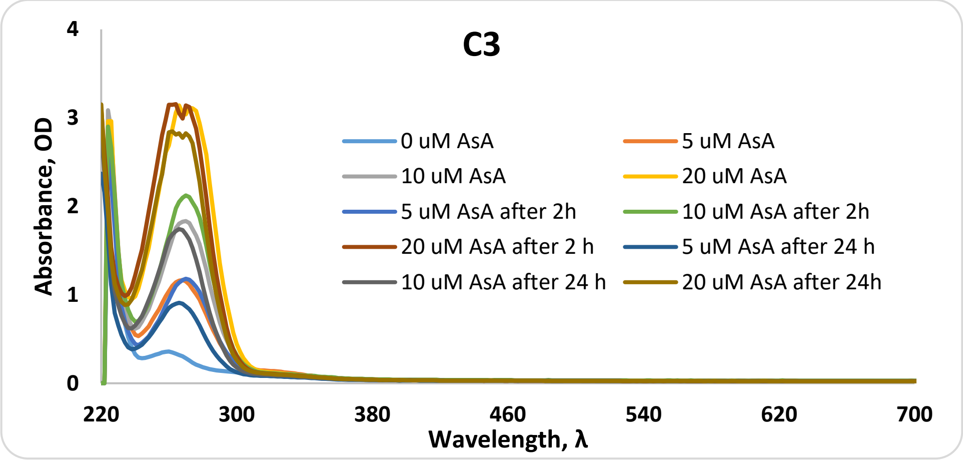
C3
Absorbance, OD
Wavelength, λ
0 uM AsA
5 uM AsA
10 uM AsA
20 uM AsA
5 uM AsA after 2h
10 uM AsA after 2h
20 uM AsA after 2 h
5 uM AsA after 24 h
10 uM AsA after 24 h
20 uM AsA after 24h

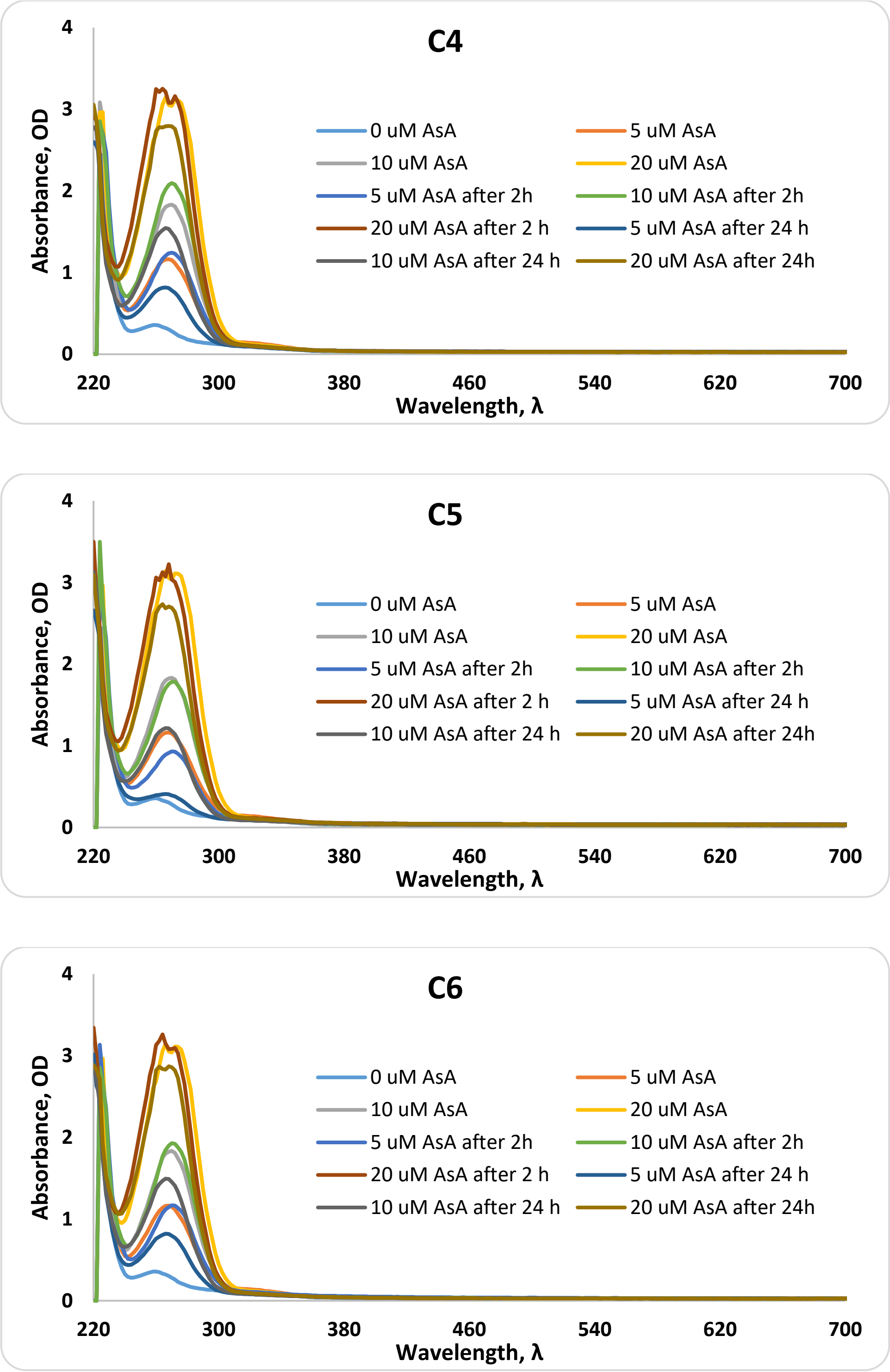


**Figure S5.** UV-Vis spectra of **C1-6** complexes recorded at different times for 24 h in PBS at 37ºC in presence of AsA (0-20 µM).

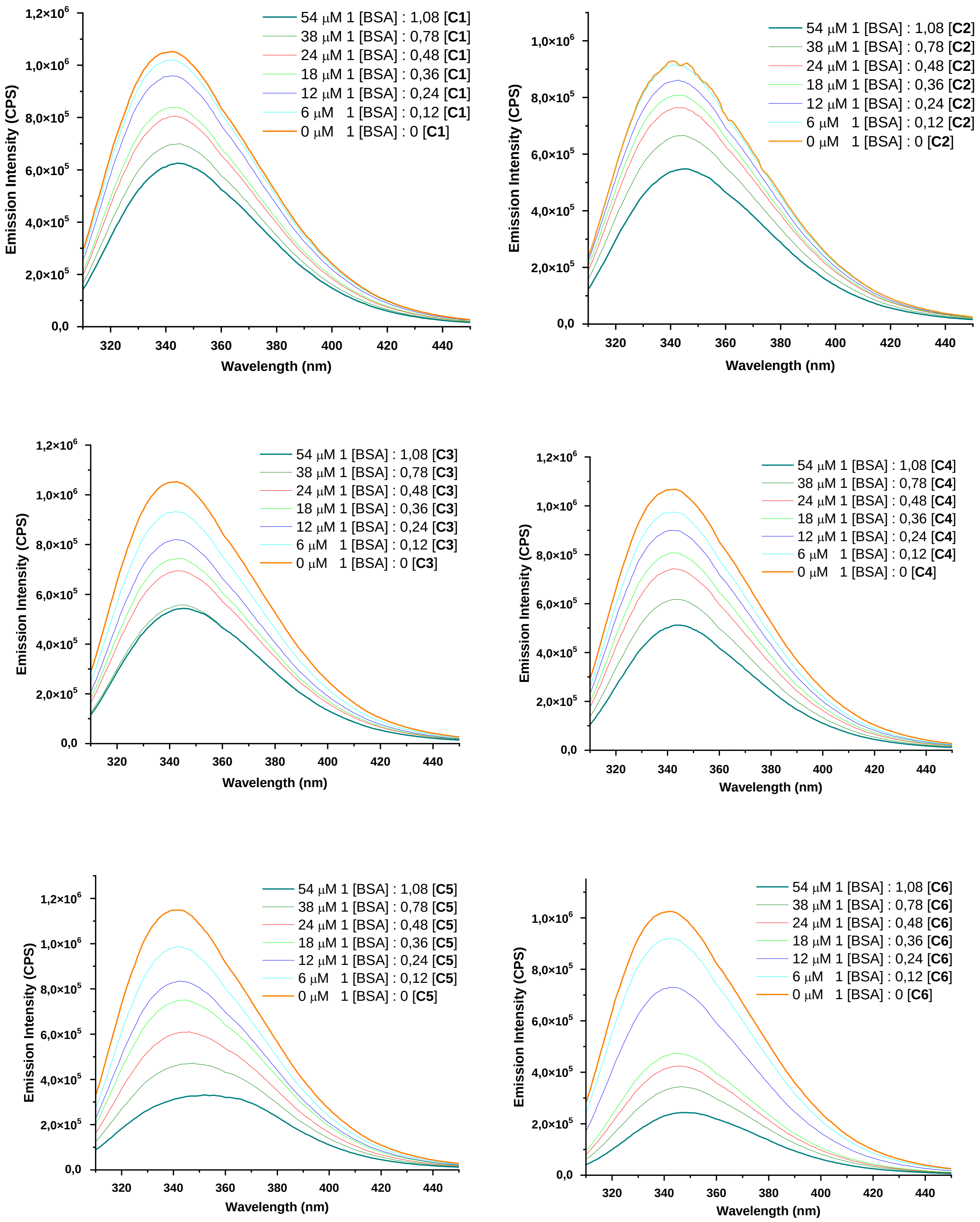


**Figure S6.** Fluorescence emission spectra of BSA at 298 K in the presence of increasing amounts of the gold(III) complexes.

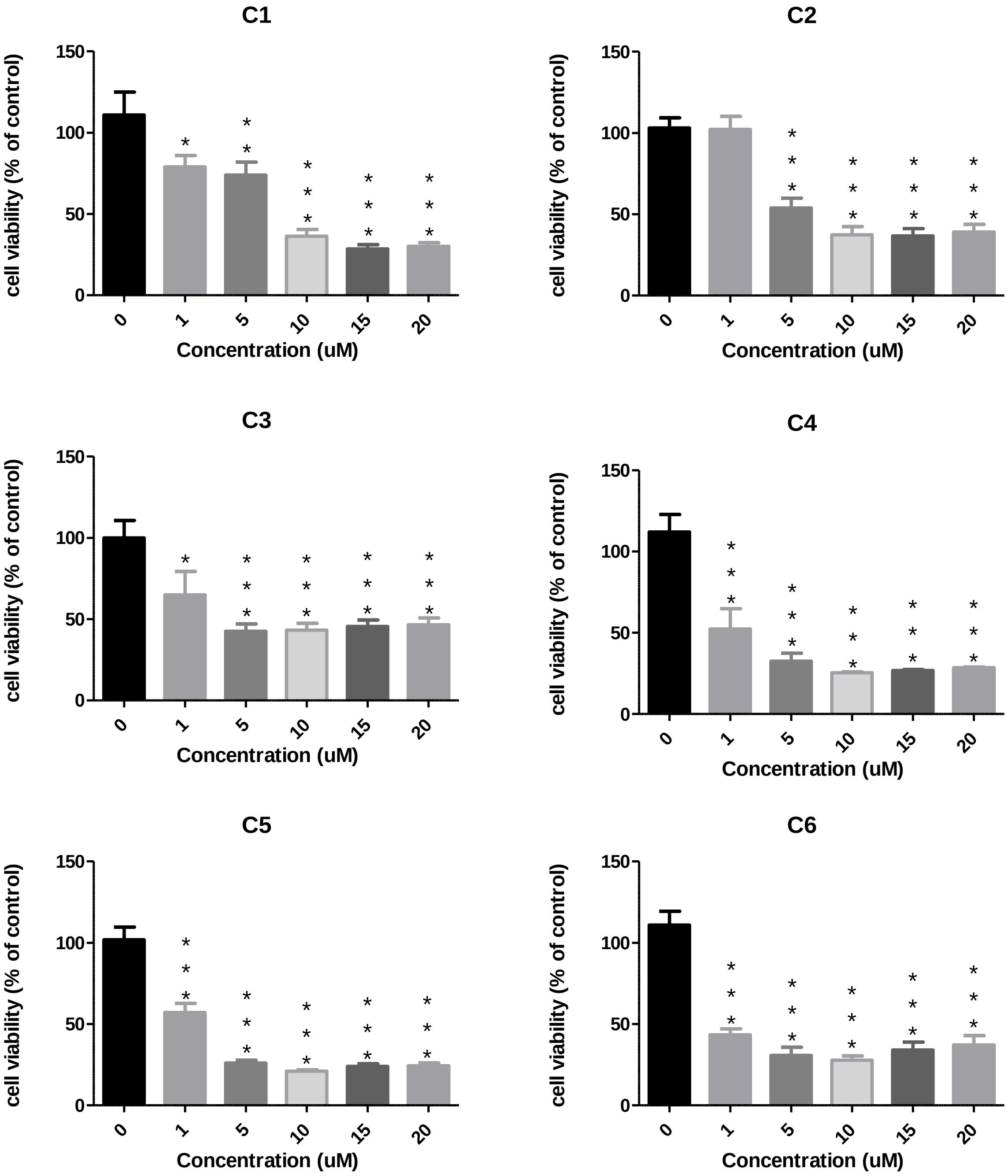


**Figure S7.** Cell viability of tumor Caco-2 cells (5 days after seeding) treated with **C1**-**6** gold(III) complexes for 72h. All the results are expressed as mean ± SEM (n ≥ 12 experiments). *P<0.05; **P<0.01; ***P<0.001 vs. control.

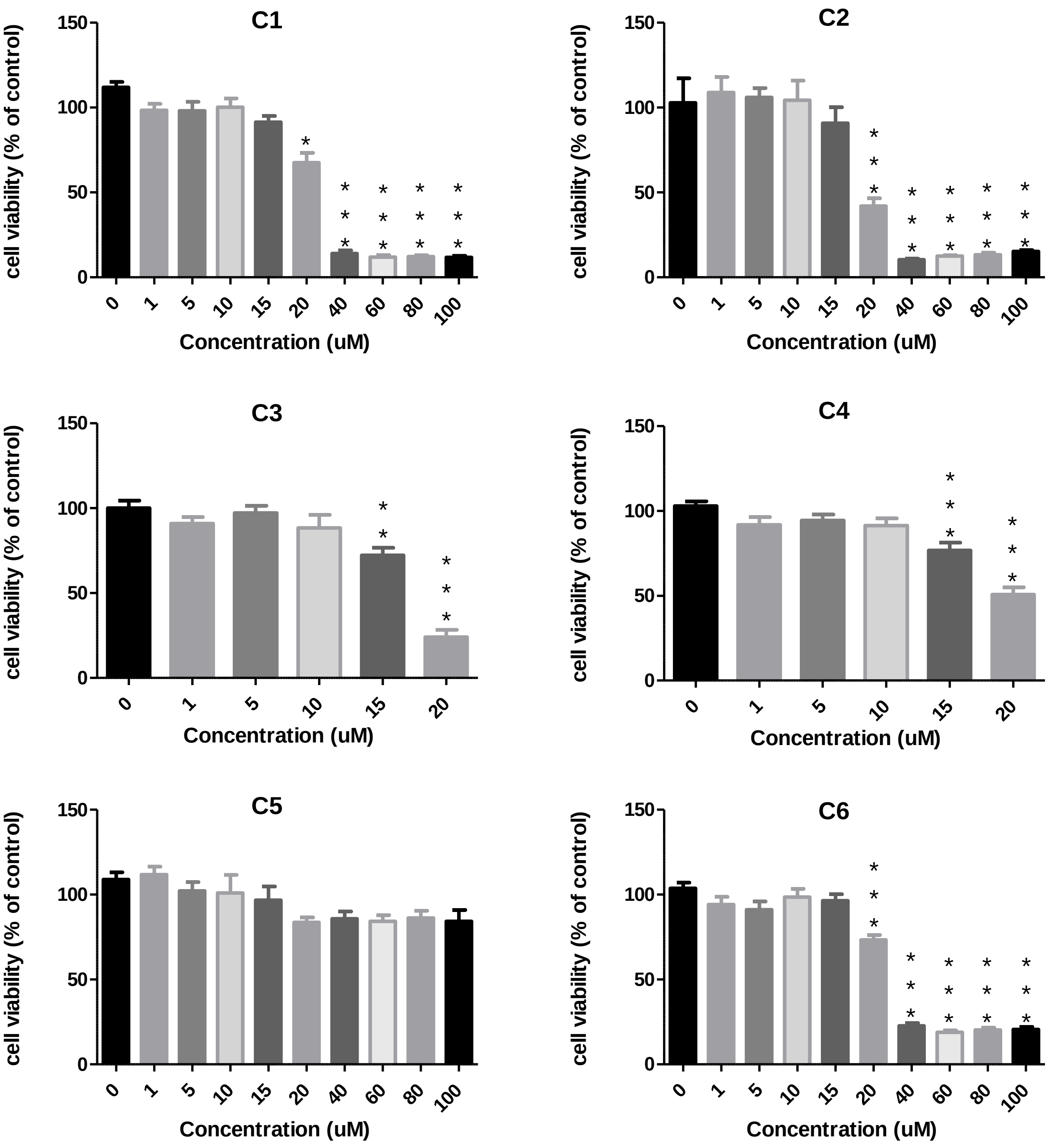


**Figure S8.** Cell viability of normal Caco-2 cells (15 days after seeding) treated with **C1**-**6** gold(III) complexes for 72h. All the results are expressed as mean ± SEM (n ≥ 12 experiments). *P<0.05; **P<0.01; ***P<0.001 vs. control.

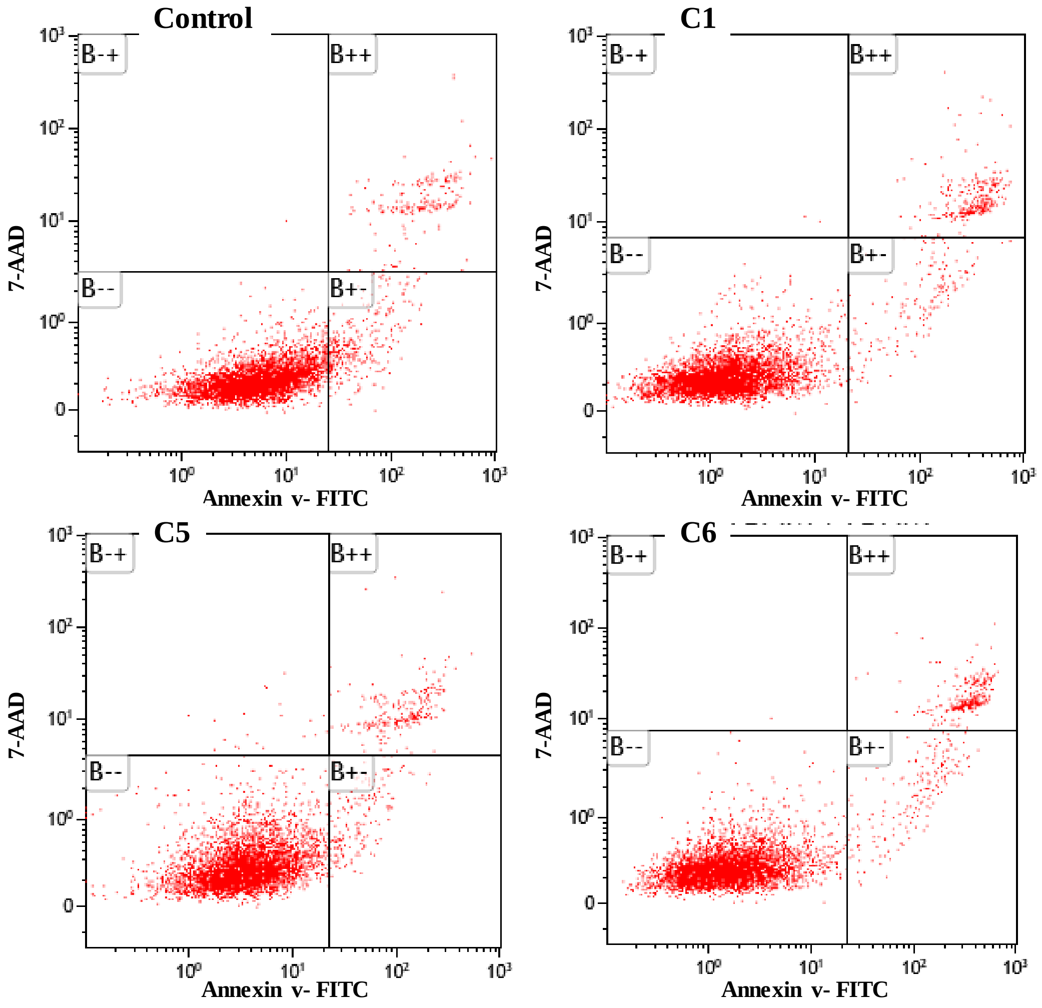


**Figure S9.** Fluorescence histograms of the distribution of cell populations in different stages: necrosis ($B^{+-}$), living cells ($B^{--}$), late apoptosis ($B^{++}$) and early apoptosis ($B^{+-}$), in control (DMSO) and gold(III) complexes (20 μM, 24 h) treated Caco-2/TC7 cells**.**

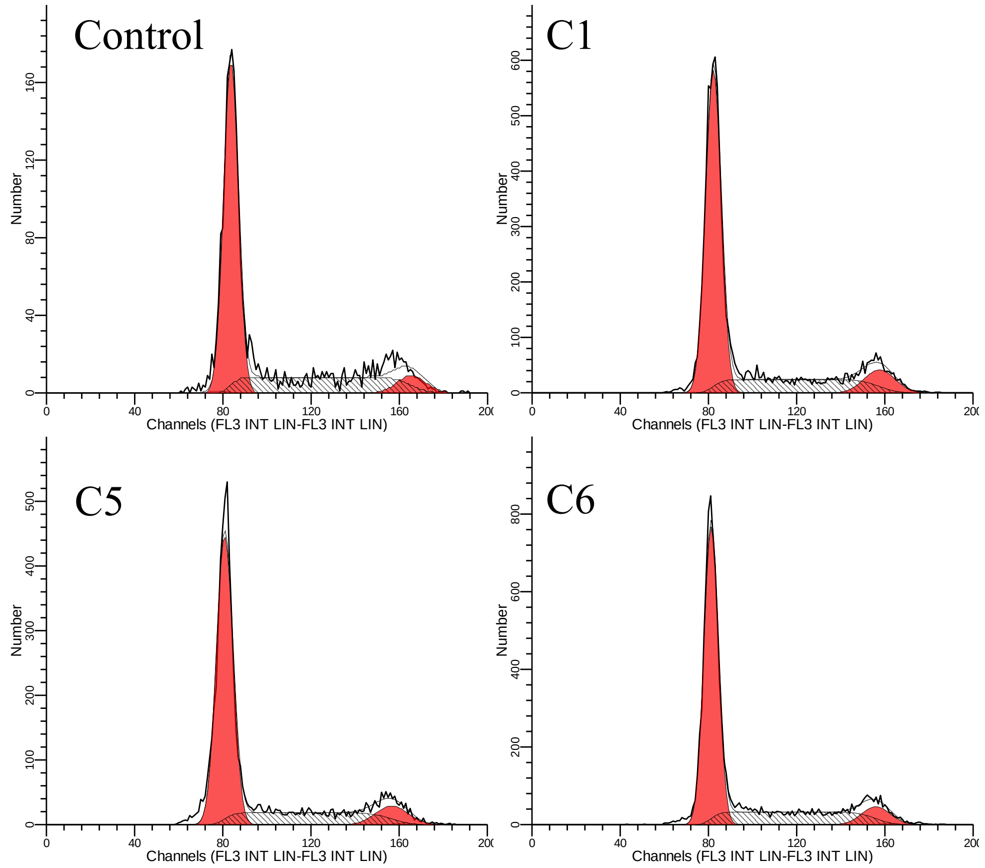


**Figure S10.** Fluorescence histograms obtained by flow cytometry of the cell populations in different phases of the cell cycle after 24 h of incubation of the cells with DMSO (control) and gold(III) complexes **1** and **5–6** (20 μM).
